# A Collaborative Approach to Treatment Strategies for Latent Tuberculosis Infection

**DOI:** 10.5888/pcd17.200124

**Published:** 2020-07-02

**Authors:** Amos Lal, Ajay Kumar Mishra, Kamal Kant Sahu

**Affiliations:** 1 Division of Pulmonary and Critical Care Medicine, Mayo Clinic, Rochester, Minnesota; 2 Department of Medicine, Saint Vincent Hospital, Worcester, Massachusetts

We read with great interest the article by Jakeman et al, “Addressing Latent Tuberculosis Infection Treatment Through a Collaborative Care Model With Community Pharmacies and a Health Department,” which addressed latent tuberculosis infection (LTBI) treatment through a collaborative approach in New Mexico (1). The authors described a new approach to aim at the ever-moving target of eradication of tuberculosis in the United States. The approach of involving community pharmacies and pharmacy visits is a brilliant idea for reaching the appropriate populations and improving health care access (2). Lal et al studied the rates of initiation and completion of LTBI treatment in a teaching hospital in Massachusetts and found that the immigrant population had a higher completion rate (93.7%) than did the population born in the United States (69.7%) (3). According to the Integrated Public Use Microdata Series, Current Population Survey, New Mexico has a large immigrant population with a potentially high prevalence of LTBI (4). It is well known that LTBI is a dormant source of tuberculosis disease in patients who are not treated. Jakeman et al carefully selected an appropriate cohort to implement their intervention.

The study by Lal et al also found that having at least 4 visits with a health care provider increased the odds of completing treatment and that the time spent during the initial visit was an important factor in completing treatment (3). We suggest that any initiative introducing a collaborative approach, such as the one described by Jakeman et al, should emphasize the importance of the initial visit. More time spent during the initial visit can potentially save time during subsequent visits and improve treatment completion rates. Although the treatment initiation and completion rates Jakeman et al achieved by using a collaborative approach are encouraging, reaching the public health goals of higher completion rates and, ultimately, disease eradication will require use of all reasonable measures.

We congratulate the authors on their work in addressing LTBI treatment. Collaborative working relationships between community pharmacies and physicians have improved patient care in many communities (2). Moving forward with an approach like the one implemented by Jakeman et al can best be achieved through better patient education and a decentralization of resources and responsibility, with a shift from large hospitals and departments of health toward community-based pharmacies and pharmacists.
